# Molecular Autonomous Pathfinder Using Deep Reinforcement Learning

**DOI:** 10.1021/acs.jpclett.4c00438

**Published:** 2024-05-09

**Authors:** Ken-ichi Nomura, Ankit Mishra, Tian Sang, Rajiv K. Kalia, Aiichiro Nakano, Priya Vashishta

**Affiliations:** Collaboratory for Advanced Computing and Simulations, University of Southern California, Los Angeles, California 90089, United States

## Abstract

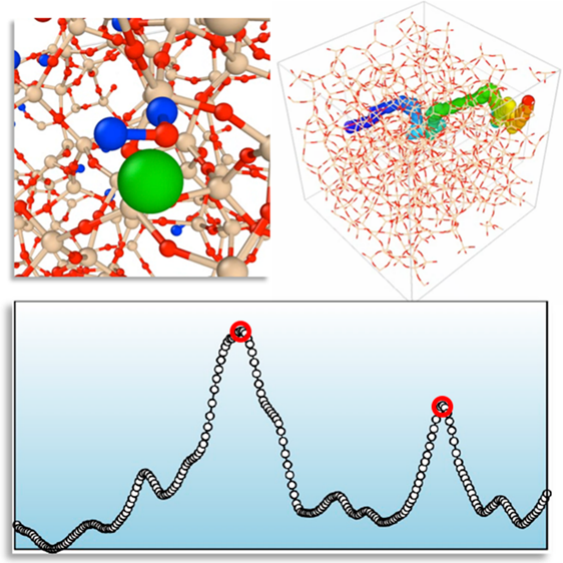

Diffusion in solids is a slow process that dictates rate-limiting processes in key chemical reactions. Unlike crystalline solids that offer well-defined diffusion pathways, the lack of similar structural motifs in amorphous or glassy materials poses great challenges in bridging the slow diffusion process and material failures. To tackle this problem, we propose an AI-guided long-term atomistic simulation approach: molecular autonomous pathfinder (MAP) framework based on deep reinforcement learning (DRL), where the RL agent is trained to uncover energy efficient diffusion pathways. We employ a Deep Q-Network architecture with distributed prioritized replay buffer, enabling fully online agent training with accelerated experience sampling by an ensemble of asynchronous agents. After training, the agents provide atomistic configurations of diffusion pathways with their energy profile. We use a piecewise nudged elastic band to refine the energy profile of the obtained pathway and the corresponding diffusion time on the basis of transition-state theory. With the MAP framework, we demonstrate atomistic diffusion mechanisms in amorphous silica with time scales comparable to experiments.

From the carburizing process of steel in the Roman age^1^ to modern industries of semiconductors,^2,3^ Li-ion batteries,^4−6^ high entropy alloys,^7,8^ and resistive memories,^9,10^ control of solid diffusion has been central in materials science and engineering. Based on the systematic study of salt diffusion in water by Graham, Fick provided the mathematical formulation of the diffusion in liquid, known as Fick’s law.^11,12^ Fick’s law describes the diffusion process as the mass transport driven by the gradient of concentration. Diffusion following Fick’s law is well-characterized by Brownian motion; however, anomalous and non-Fickian diffusions at nanoscale have been attracting great attention to further advance the frontier of nanoengineering and novel system designs.^13,14^

Molecular dynamics (MD) simulation is an excellent computational tool that may provide mechanistic understandings of atomistic-level events; at the same time, it poses great scientific challenges. Diffusion in solids is a slow process in general. The computational cost of MD simulations prohibits accessing the relevant time scale of molecular diffusion in solids. The current state-of-the-art (SOTA) direct MD simulation achieves a million-atom simulation for over 100 μs per day,^15^ which amounts to a remarkable spatiotemporal throughput of *NT* = 100 where *N* is the total number of atoms and *T* is the simulation time. This allows access to many biologically relevant processes; however, it is still intractable to simulate the hours- or days-long time scales that are commonly observed in solids. Here, we propose the molecular autonomous pathfinder (MAP) framework combining deep reinforcement learning (DRL)^16−18^ and the transition-state theory (TST)^19^ to explore complex energy landscapes and automatically uncover energy-efficient diffusion pathways with minimal human intervention. As a demonstration of long-time atomistic simulation, we apply the MAP framework to a decades-long problem of the water diffusion process in silica glass.

Great strides have been made in the last two decades to extend accessible time scale using MD simulation,^20^ including accelerated molecular dynamics (AMD) simulation methods such as temperature-accelerated dynamics, parallel replica, and hyperdynamics. While such physically accurate exploration of the energy landscape is necessary to quantify long-time dynamics, its intractable exponential complexity^21^ warrants an alternative heuristic to serve much needed technological needs for quickly screening resilient materials against failure. To estimate the time-to-failure of materials, the long-tail behavior of the probability distribution function plays a crucial role. For example, the reliability and lifetime modeling commonly employ a Weibull distribution. Generalized extreme value distribution^22^ concerns the distribution of block maxima to deal with events significantly deviating from their mean, such as catastrophic failures. The scope of the MAP framework therefore is to present the existence of possible diffusion pathways, with an upper bound estimate of diffusion time, which are critical inputs for materials lifetime analysis and reliability tests. Further discussion is given below in the description of the MAP framework.

Reinforcement learning^23^ is a field of machine learning in which an agent interacts with the environment to find an optimal policy by maximizing the cumulative reward. DRL extends conventional RL algorithms using deep learning techniques and finds diverse applications in robotics, video games, finance, and healthcare, as well as a mission-critical plasma control in the nuclear fusion reactor.^24^ DRL has been also applied to molecular simulations, including the optimization of advanced materials synthesis,^25,26^ novel drug designs,^27,28^ and transition state (TS) search.^29^ Deep Q-Network (DQN) is one of the most successful DRL algorithms based on Q-learning,^30^ which solves the Bellman optimality equation
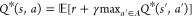
1where *s* and *s*′ are the current and next states, *a* is the action, γ is the discount factor, *r* is the reward, Q* is the optimal state-action function, and  denotes an expectation value.

Using DQN, Minh et al. demonstrated superhuman performance for 49 Atari games by using only pixels and game score as inputs. The Q-function is modeled as a convolutional neural network (CNN) to incorporate the complex state definition, *i.e*., pixel images of the Atari games. The network parameter is trained by minimizing the temporal-difference (TD) error as loss function given as
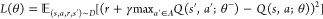
2where agent’s experience *e*_*t*_ = (*s*_*t*_, *a*_*t*_, *r*_*t*_, *s*_*t*+1_) is randomly selected from the experience replay buffer *D*. The Q-function is modeled as a three-dimensional CNN. θ^–^ and θ are the parameters of the target and behavioral networks to reduce the variance during model training, respectively. Several extensions to the vanilla DQN have been developed to further improve the performance. Hessel et al. proposed Rainbow DQN and achieved roughly 2× improvement for all 57 Atari games.^31^ Horgan et al. demonstrated that distributed training could also significantly improve the performance.^32^ Their distributed DRL algorithm consists of a single learner process that learns from experiences collected by hundreds of actor processes. These experiences are prioritized by their TD error value; thereby, the learner can avoid experiences that have already been learned well. With the distributed replay buffer and using 360 actors, they have achieved approximately 2.3× performance improvement in about half of the SOTA time.^32^

Inspired by the DRL architecture that offers superhuman capability to explore an extremely large parameter space and autonomously develop an optimal policy, we have designed the MAP architecture as shown in Figure 1. In the first phase, we use a scalable DRL to train autonomous molecular agents to find energy-efficient diffusion pathways. While offline training takes advantage of precomputed data it suffers from the distribution shift problem, which is one of the central challenges in offline RL.^4^ On the other hand, the application of online training is severely limited by its sample inefficiency. To realize a generic framework that is applicable to a wide class of material problems, MAP employs online training to avoid the distribution shift problem with accelerated sampling efficiency using distributed and asynchronous agents (see Figure S1). In the second phase of MAP, the obtained diffusion pathways are divided into mutually exclusive segments, each of which contains a candidate TS. We apply a piecewise nudged elastic band (pNEB) method on each segment to refine the energy barriers. Based on transition-state theory,^33^ the associated time of each segment is estimated as
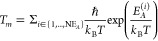
3where ℏ is the reduced Plank constant, *k*_B_ is Boltzmann constant, *T* is temperature, NE_*A*_ is the total number of energy barriers, and *E*_*A*_^(*i*)^ is the *i*th energy barrier along the pathway. Since each NEB calculation can be done in parallel, our MAP framework efficiently evaluates the total diffusion time.

**Figure 1:**
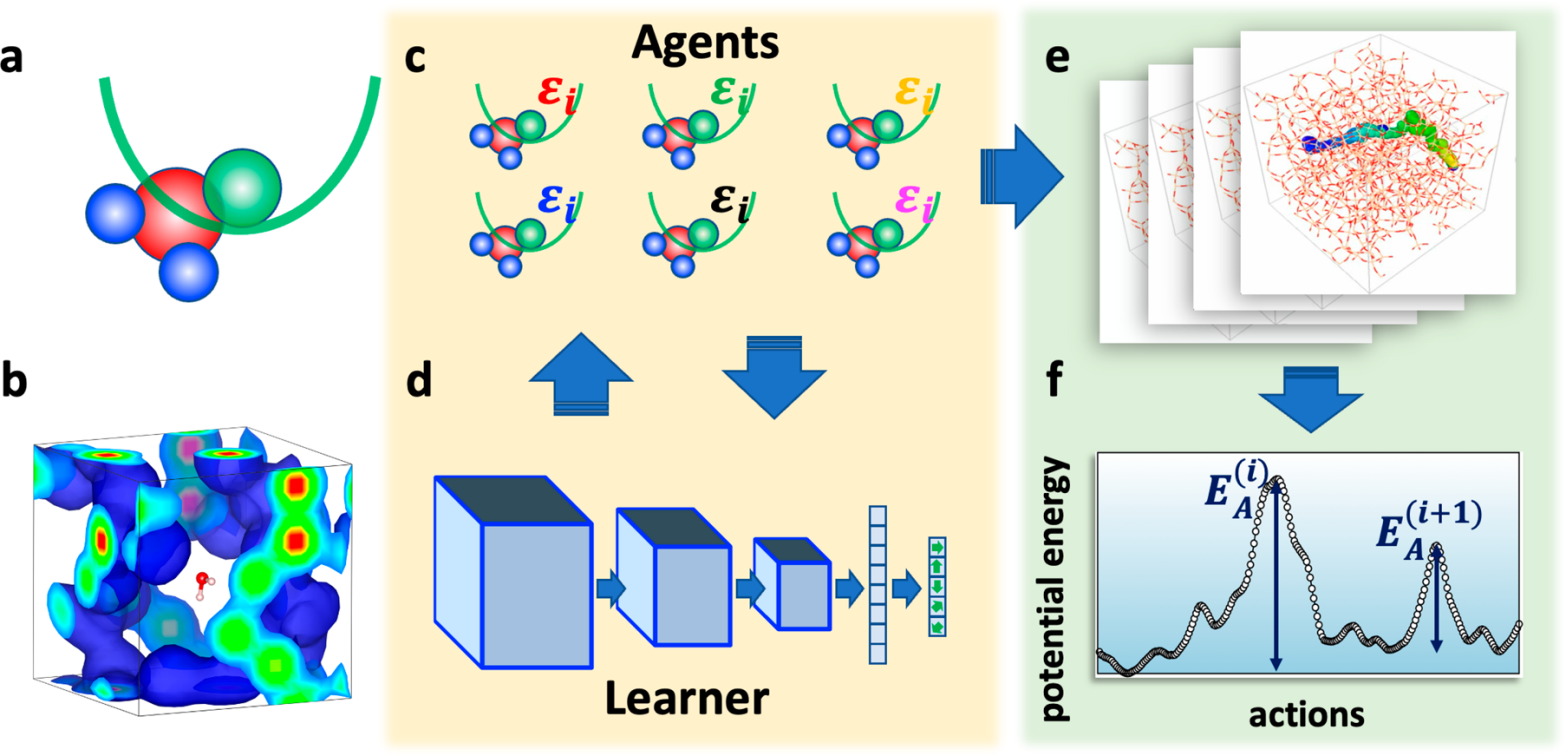
Molecular autonomous pathfinder framework. (a) A schematic of agent (green) navigating a molecule in the simulation system. RL agent and target molecule are connected by a harmonic potential interaction indicated by the green curve. (b) A snapshot of a state. Three-dimensional grid captures the local density distribution of surrounding atoms around the target molecule. The value of the 3D grids is the sum of the Gaussian kernel centered at the neighbor atoms. (c and d) A schematic of agents and learner processes. Note that each agent has its own environment and does not interact with other agents. When an agent is instantiated, they are assigned different ε values (indicated by *ε*_*i*_) making some agents favor Q-value predicted by the CNN model (exploitation) and others behave more randomly (exploration). Agents’ experiences are sent to the learner process and prioritized with their TD error value. Learner trains three-dimensional CNN that maps the state to the Q-function value for each action by minimizing the TD error. (e and f) Obtained diffusion pathways are further refined using the piecewise-NEB algorithm for an accurate description of transition states.

Water diffusion in silica is a crucial process in predicting and controlling the mechanical response of silicate materials. The presence of water is known to affect the physical and chemical properties of silicates significantly; however, its atomistic mechanism has been argued for many decades.^34−41^ Stress corrosion cracking (SCC) is an archetypal example where subcritical crack growth is observed under a moist environment. With moisture, a crack tip of silica glass is found filled with water. These water molecules react with stretched siloxane bonds and break into two silanol groups subjected to tensile loading. A three-stage model is usually used to describe the overall fracture behavior. While a simple mechanical argument applies to the first and third stages, the crack growth rate does not depend on applied stress in the second stage where molecular diffusion is considered as the rate-limiting step. While a conventional view of SCC is sequential SiO bond breaking by water at the crack tip, recent studies have shown the possibility of fast diffusion pathways mediated by nonbridging oxygens (NBOs) as well as the increased free volume due to stress concentration around the vicinity of the crack tip. Water diffusion in silica glass also finds important applications in earth and planetary sciences.^42^ To investigate molecular diffusion mechanisms through the mantle, silica glass has been used as an experimental platform.^35,36^

Since the learning of RL agents is driven solely by the reward functions that are hand-tuned hyperparameters, care needs to be taken on how to interpret the obtained RL trajectories. Furthermore, while learning metrics increase over time, it does not necessarily mean that the obtained trajectory has converged to the most energy-efficient pathway within a given reward structure, computing resource, and training time. These trajectories that the MAP framework generates should be considered as a set of possible diffusion pathways with an upper bound estimate of diffusion time. As such, the MAP framework may provide the long-tail samples in a reliability test of material service lifetime.^43,44^

The following section describes the key components of the MAP framework.

*Environment*. The environment is modeled by the reactive molecular dynamics (RMD) simulation. To incorporate the energetics of bond breaking and formation during agent training, we employ the quantum-mechanically validated ReaxFF^45,46^ force field and a scalable MD software RXMD.^47^ The silica glass structure was created by the melt–quench method.^48^ The system dimensions are (30.48 Å)^3^. The total number of atoms are 1,593 including one H_2_O molecule navigated by the RL agent. Throughout the training, the system is thermalized at 10 K with the canonical (*NVT*) ensemble with a time step of 0.5 fs.

*Agent*. An agent is defined as a harmonic interaction function that is bound to an atom to facilitate molecular diffusion through silica glass (Figures 1a and S1). The center of the harmonic potential, i.e., the agent position, is bound to the O atom of the H_2_O molecule and updated following the agent’s action. We employ discrete actions defined as displacement vector, *a⃗*, to update the agent’s position. We choose *a⃗* = [(1,0,0), (0,1,0), (0,0,1), (0,–1,0), (0,0,–1)] to avoid the agent oscillation problem.^49^ State, *s*, is defined as a three-dimensional grid that approximates the distribution of neighboring atoms around the agent. From each neighbor atom, the Gaussian kernel is used to represent their contribution to the local density. Based on the predicted Q-function value given a state *s*, the agent updates its position **r**_agent_ by a chosen vector multiplied by a displacement magnitude δ.
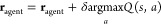
4To facilitate the agents’ exploration, we use ε-greedy policy where an action is randomly chosen with the probability of ε value regardless of the Q-value. For the distributed training, each agent is assigned a different randomness factor ϵ = (0, ϵ_max_) to control their extent of exploration (Figure 1c). After an action has been taken, the H_2_O molecule and silica system are relaxed by a short RMD simulation.

*Learner*. The main tasks of the learner process are to organize agents’ experiences by their TD error, update model parameters of the Q-function, and synchronize the updated model with agents. When the learner receives the agent’s experience, the learner computes the TD error and stores a tuple of experience and the computed TD error in the replay buffer. To compute the loss function eq 2, the learner randomly samples batch-sized tuples from the reply buffer with a probability proportional to the TD error. A large TD error value indicates an unearned situation for the model. The prioritized reply buffer is particularly useful when many agents tend to accumulate experiences around the initial starting location.^32^

*Reward*. We used five reward functions in total to train the agents. The function *R*_position_ gives reward based on the distance between a predefined goal and the location of the agent. In this study, we used the *x*-coordinate of the agent as the reward value. This monotonically increasing function serves as the baseline of the overall reward structure. *R*_energy_ rewards an agent if the current state has a potential energy lower than the reference potential energy. To minimize the noise in the reference energy, we use the mean of the potential energy over a prescribed number of MD steps. *R*_density_ penalizes the agent, i.e., gives a negative reward if the distance between the agent and surrounding atoms becomes too small. Similarly, we also apply a penalty *R*_distance_ if the distance between the agent and the water molecule becomes greater than a prescribed threshold. Technically, an agent may earn rewards by making actions with positive rewards without moving away from the same location. To achieve efficient learning of the environment, a common practice is to apply a time penalty, *R*_time_, with which an agent receives a negative reward whenever a new action is made. Total reward *R*_total_ is the sum of the five rewards with weighting perfectors that are tuned as hyperparameters.

Given the reward structure, we investigated the training efficiency as a function of the number of agents, *N*. As the measure of the agent’s performance, we record the agent’s *x*-coordinate and total reward at the end of each epoch. Figure 2 shows a typical agent’s performance with *N* = 1, 4, and 16 during 48 h of training. Overall, increasing the number of agents results in better performance for both the agent’s final coordinate and the total reward. With *N* = 1, the total reward is kept low, although the final position consistently increases. With *N* = 4, both metrics increase at the beginning; however, the increase rate slows down after 10,000 steps. With *N* = 16, the final agent position increases further with a smaller deviation. While the final position is comparable with *N* = 4 and 16 up to 10,000 steps, the total reward with *N* = 16 is noticeably better, approximately 2 times greater than the one with *N* = 4.

**Figure 2:**
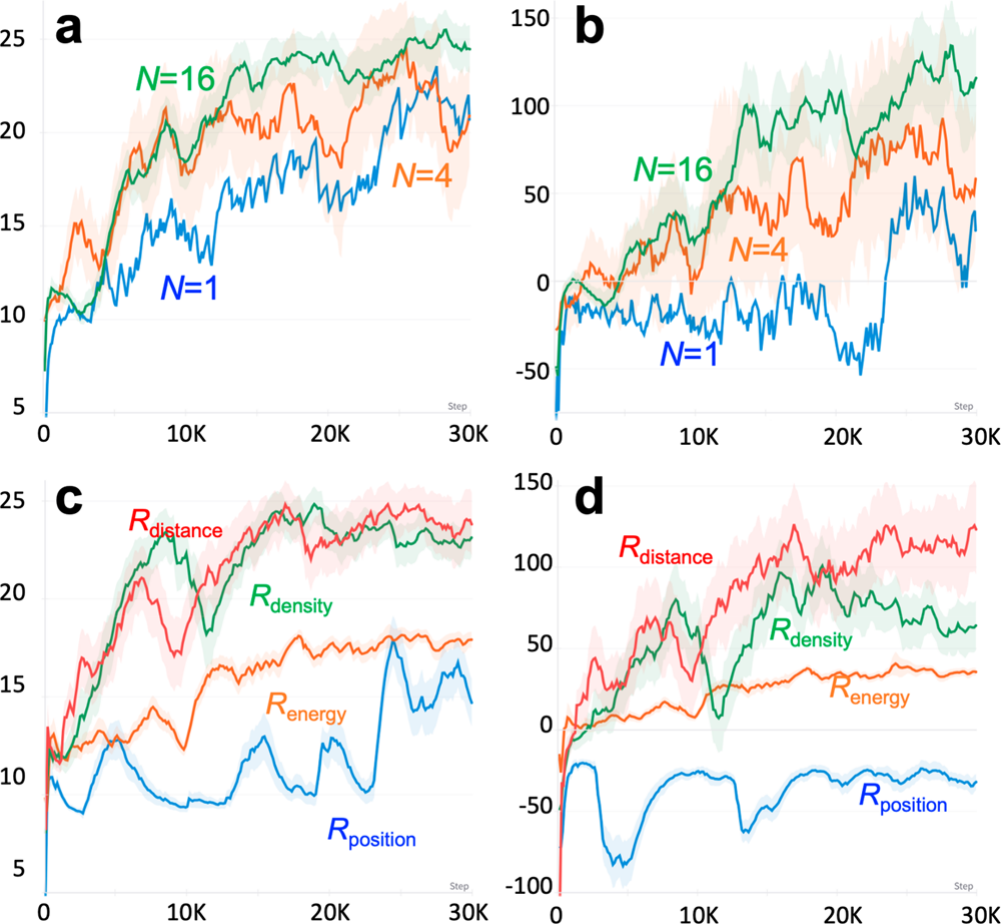
(a and b) Training performance as a function of the number of agents. Panels a and b show the agent’s final position and the total reward with *N* = 1, 4, and 16, respectively. For the training with *N* > 1, the solid line shows the mean of all agents’ performance and the shade represents their standard deviation. Overall, the training performance improved by increasing *N*. While the final agent position is comparable with *N* = 4 and 16 up to 10,000 steps, the total reward with *N* = 16 is approximately 2 times greater than the one with *N* = 4, indicating that more efficient pathways have been discovered with *N* = 16. (c and d) An ablation study on the reward functions where the agent’s final position and total reward are monitored with one of the four rewards (*R*_position_, *R*_energy_, *R*_density_, and *R*_distance_) are turned off. All results are obtained with *N* = 16. *R*_position_ serves the baseline reward while *R*_energy_ helps agents find diffusion pathways with greater total rewards.

Next, we performed an ablation study with one of the reward functions turned off (Figure 2c,d). We observe that *R*_position_ serves as the baseline reward for an agent to learn the proper direction to move. With *R*_position_ off, the agent’s final position remains around 13 Å (out of 30.48 Å) and the total reward remains negative. With *R*_energy_ turned off, the agent also appears to struggle, resulting in a suboptimal final position around 18 Å and the value of total reward is about 30, respectively. On the other hand, a decent training performance is obtained with either *R*_density_ or *R*_distance_ turned off, signifying the important contribution of *R*_position_ and *R*_energy_ for agents to learn the environment.

Once sufficient pathways have been sampled, we applied pNEB to refine the description of transition states and used eq 3 to estimate the diffusion time. Figure 3 shows the potential energy profile of the top six episodes (episodes I–VI) ranked by their diffusion time after 48 h of training using *N* = 16. The original energy profile obtained by the RL agent is divided into several segments, each of which contains one initial, final, and a candidate of TS. Subsequently, pNEB is simultaneously performed on each segment in the energy profile. After the pNEB calculation, the five episodes (II–VI) have converged to a similar energy profile, in which a rather flat region continues up to around the 90th action, followed by two noticeable energy barriers around the 100th and 120th actions. In addition to the existing two barriers, an additional TS with a relatively small activation energy (∼ 0.8 eV) is observed at the 117th action in episode I.

**Figure 3:**
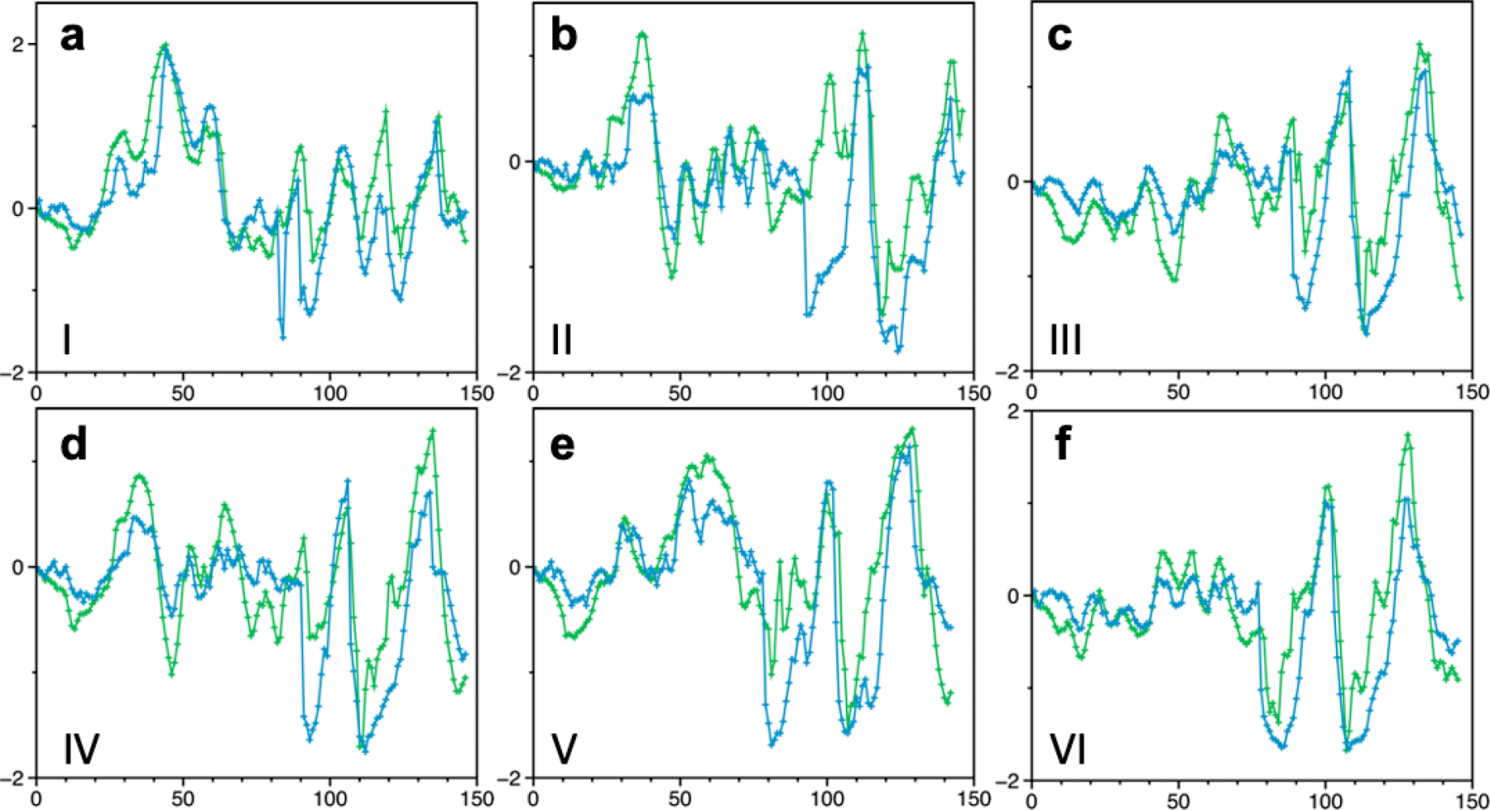
(a–f) Potential energy profile before (green) and after (blue) pNEB on the last six energy efficient pathways using *N* = 16 after 48 h of training. The vertical axis is the potential energy of the system in eV units, and the horizontal axis is the number of actions. The cumulated number of actions of the episode I–VI is 31,068, 30,752, 28,243, 27,835, 24,020, and 23,989, respectively. The initial, final, and transition states in the original energy profile are identified by SciPy signal processing library. The five episodes except episode I have converged to a similar profile that consists of an initially flat region followed by two consecutive energy barriers approximately around the 100th and 120th action. Episode I shows an additional peak around the 120th action.

To obtain atomistic insights along the diffusion pathway, Figure 4 presents a series of snapshots of the TSs of episode I shown in Figure 3a. In the episode, the H_2_O molecule is first weakly absorbed by a siloxane bond^50^ and detaches from the site after a few actions. After the 80th action, the H_2_O molecule is moved to an undercoordinated Si site, which results in the reduction of potential energy approximately by 1 eV. Soon after, the H_2_O molecule hops to another undercoordinated Si site by a concerted switching of neighboring Si atoms (Figure 4c). Finally, the H_2_O molecule detaches from the silicon, labeled as Si3 in Figure 4d. In episode I, however, the hopping between the two Si atoms occurs in two steps (shown in Figure 4e,f). Instead of the direct hop, the H_2_O molecule approaches Si3 moving around Si2, accumulating the strain energy by distorting the silica network. The acquired strain energy facilitates the desorption of the H_2_O molecule from the Si2 site, reducing the overall energy barrier. Consequently, the estimated diffusion time by the two-step hopping mechanism is reduced by a factor of 15, namely 0.124 vs 1.93 days to travel 3 nm at 350 °C in episodes I and II, respectively. The obtained time scale is comparable to experimental observations.^36,38^

**Figure 4:**
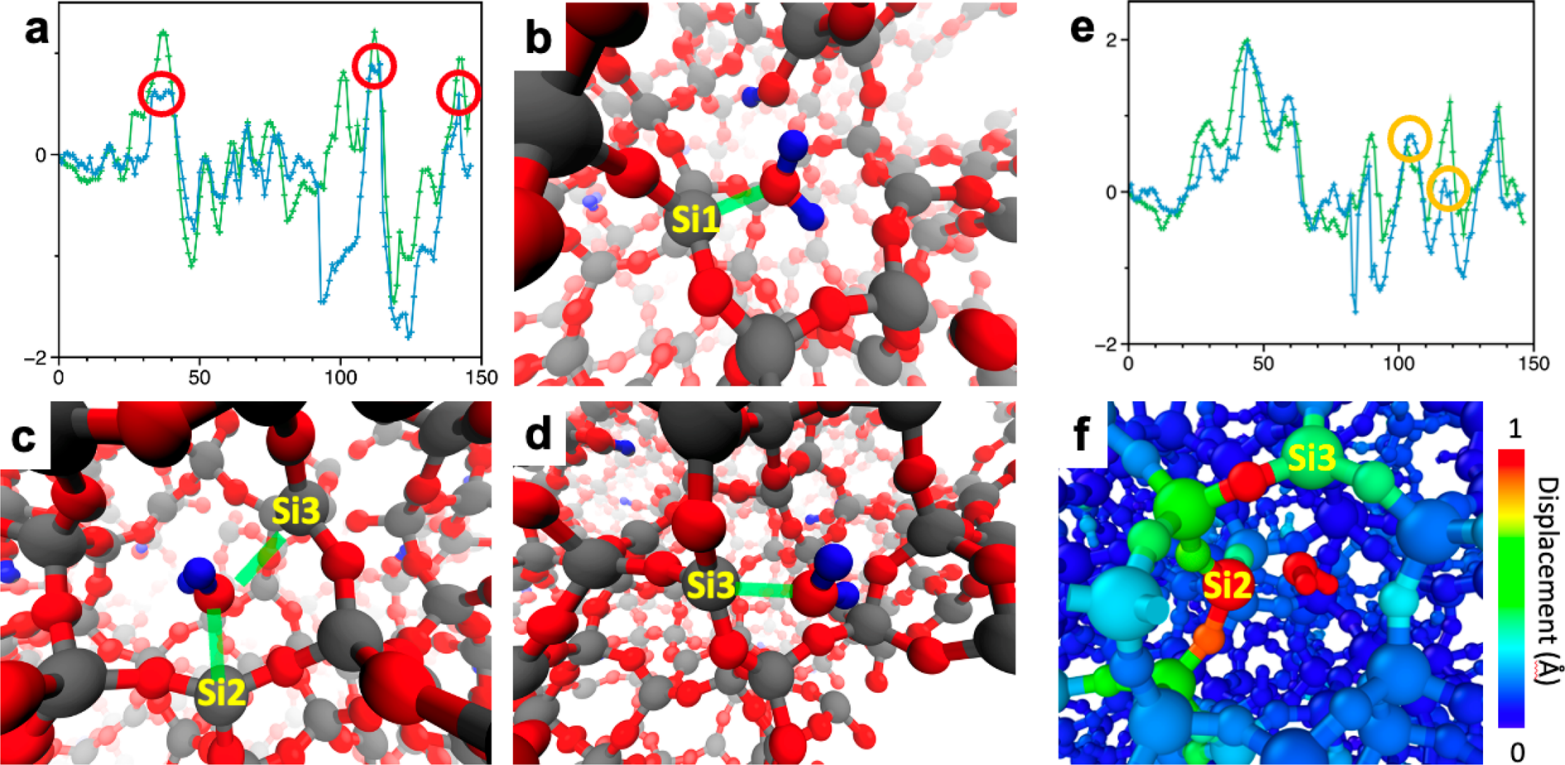
(a) Potential energy profile before (green) and after (blue) pNEB and three TSs (red circles) in episode II. (b–d) Atomic configurations of the three TSs in panel a. Green lines in panels b–d indicate the closest Si from the O atom in the RL-guided H_2_O molecule. (e) Potential energy profile of episode I before (green) and after (blue) pNEB along with the two TSs (orange circles) during the H_2_O molecule hopping from Si2 and Si3. (f) Atomic configuration of the second TS color-coded by the atomic displacement from their initial position. A large displacement (over 1 Å) on Si2 indicates the stored mechanical strain energy in the TS.

In conclusion, we have developed a scalable AI-guided framework combining DRL and TST to study the long-term diffusion process in solids. Obtained energy profiles are further refined using piecewise NEB to efficiently translate to the overall diffusion time. The design of the MAP framework focuses on its transferability to a wide class of material problems; for example, the use of online learning to eliminate the necessity of generating training data sets that often requires expert domain knowledge, and the highly efficient sampling of agent experiences by taking advantage of advanced computing architectures. We applied the MAP framework to a long-standing problem of water diffusion in silica glass, which revealed a strain-assisted diffusion pathway. In addition to the inorganic silica glass presented in this study, the MAP framework has been successfully applied to organic polymer systems,^51^ providing a novel approach to investigate long-time diffusion mechanisms with atomistic-level insights.

## Supporting Information Available

Additional information on algorithm scalability, RMD system setup and simulation details, and the definitions of state and Q-function (PDF)
Example of obtained molecular diffusion trajectory: Green sphere as RL agent binding the O atom of an H_2_O molecule (MOV)

## Supplementary Material

jz4c00438_si_001.pdf

jz4c00438_si_002.mov
