# Supplementary material for: Molecular Autonomous Pathfinder Using Deep Reinforcement Learning
Source: J Phys Chem Lett. 2024 May 9;15(19):5288–94. doi: 10.1021/acs.jpclett.4c00438 (PMC11103691; doi:10.1021/acs.jpclett.4c00438)
Supplement: Supplementary file 1 — jz4c00438_si_001.pdf [file jz4c00438_si_001.pdf]

Supporting Information for "Molecular Autonomous Pathfinder using Deep Reinforcement Learning."

Ken-ichi Nomura, Ankit Mishra, Tian Sang, Rajiv K. Kalia, Aiichiro Nakano, and Priya Vashishta

Collaboratory for Advanced Computing and Simulations, University of Southern California, Los Angeles, CA 90089, USA

1. Accelerated Online Learning and Sample Efficiency via Distributed Asynchronous Agents

We use Ray library, a scalable python library for distributed AI/ML framework [1], to implement MAP framework. To realize a fully online training framework and distributed agents that are asynchronously collecting their experiences, the learner and agent processes communicate through a shared queue via Ray library. Fig. S1 shows the size of replay buffer, *i.e*. the number of accumulated agents' experiences in the shared queue, with the number of agents $N = 1$, 4, and 16 with the sampling rate of 0.515, 2.256, and 9.03 experiences/step, respectively. MAP framework shows a superlinear scaling in the sample efficiency, namely 17.64 times speed up with $N = 16$ over the baseline of a single agent with $N = 1$.

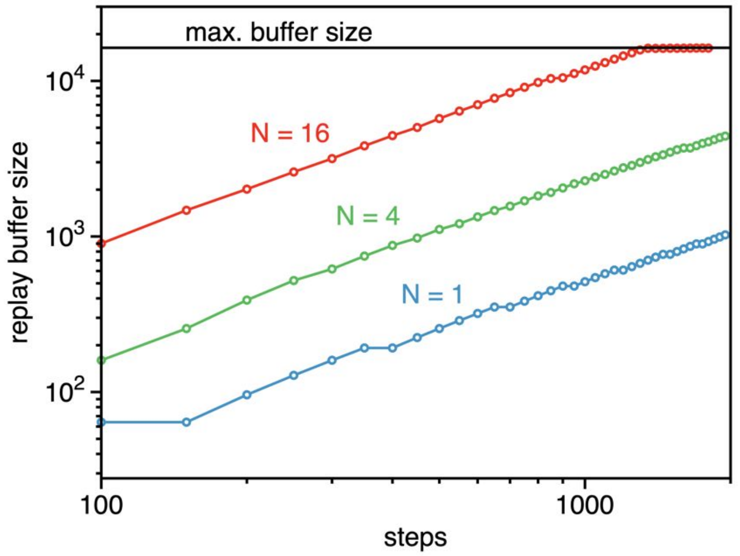

Fig. S1: The size of the replay buffer with respect to the training step. The maximum replay buffer size is set as 16,384.

2. Reactive Molecular Dynamics (RMD) Simulation of Silica Water System.

The amorphous silica system was created by a melt-quench method. After a pure silica glass system is created, the undercoordinated Si and O atoms (i.e. defect atoms) are terminated with H atom. The simulation system dimensions are (30.48Å)^3, which contains 512 Si atoms, 1,025 O

atoms, and 56 H atoms, including one H_2O molecule guided by RL agent. All RMD simulations are performed using RXMD software[2] with a ReaxFF force field [3] developed for amorphous silicates with water to investigate water and proton diffusion through the system [4]. The force field parameters have been fitted to reproduce the DFT-level accuracy of energetically favorable diffuse pathways for an ion through a silicate system [4].

3. Definitions of State and Q-function.

The state is defined as a three-dimensional grid that represents the local atomic density around the agent's location within a cutoff distance of 5 Å. The voxel resolution is 0.3Å totaling $33 \times 33 \times 33$ grids. Gaussian Kernel is used to compute the density contribution from each neighbor atom. A three-dimensional CNN is used to model the Q-function. The network consists of three convolutional (conv) layers followed by two fully connected (FC) layers. For each conv layer, kernel size and strides are (8, 2), (4, 1) and (3, 1), respectively. We use with ReLu activation function between the conv layers. Each conv layer uses 32, 64, and 128 channels and the first and second FC layers have 512 and 80 nodes, respectively.

References

(1) Moritz, P.; Nishihara, R.; Wang, S.; Tumanov, A.; Liaw, R.; Liang, E.; Elibol, M.; Yang, Z.; Paul, W.; Jordan, M. I. Ray: A distributed framework for emerging {AI} applications. In *13th USENIX symposium on operating systems design and implementation (OSDI 18)*, 2018; pp 561-577.

(2) Nomura, K.; Kalia, R. K.; Nakano, A.; Rajak, P.; Vashishta, P. RXMD: A scalable reactive molecular dynamics simulator for optimized time-to-solution. *SoftwareX* **2020**, *11*, 100389.

(3) Senftle, T. P.; Hong, S.; Islam, M. M.; Kylasa, S. B.; Zheng, Y.; Shin, Y. K.; Junkermeier, C.; Engel-Herbert, R.; Janik, M. J.; Aktulga, H. M. The ReaxFF reactive force-field: development, applications and future directions. *npj Computational Materials* **2016**, *2* (1), 1-14.

(4) Hahn, S. H.; Rimsza, J.; Criscenti, L.; Sun, W.; Deng, L.; Du, J.; Liang, T.; Sinnott, S. B.; Van Duin, A. C. Development of a ReaxFF reactive force field for NaSiO x/water systems and its application to sodium and proton self-diffusion. *The Journal of Physical Chemistry C* **2018**, *122* (34), 19613-19624.
